# *Waxy* is an important factor for grain fissure resistance and head rice yield as revealed by a genome-wide association study

**DOI:** 10.1093/jxb/erac330

**Published:** 2022-09-02

**Authors:** Zhuyun Deng, Yuxia Liu, Chunyan Gong, Bingtang Chen, Tai Wang

**Affiliations:** Key Laboratory of Plant Molecular Physiology, Institute of Botany, Chinese Academy of Sciences, Beijing 100093, China; Key Laboratory of Plant Molecular Physiology, Institute of Botany, Chinese Academy of Sciences, Beijing 100093, China; University of Chinese Academy of Sciences, Beijing 100049, China; Key Laboratory of Plant Molecular Physiology, Institute of Botany, Chinese Academy of Sciences, Beijing 100093, China; Key Laboratory of Plant Molecular Physiology, Institute of Botany, Chinese Academy of Sciences, Beijing 100093, China; Key Laboratory of Plant Molecular Physiology, Institute of Botany, Chinese Academy of Sciences, Beijing 100093, China; University of Chinese Academy of Sciences, Beijing 100049, China; Innovation Academy for Seed Design, Chinese Academy of Sciences, Beijing 100101, China; CSIRO Agriculture and Food, Australia

**Keywords:** Amylose, genetic variations, genome-wide association study, grain fissure resistance, head rice yield, near-isogenic lines, *Oryza sativa*, rice germplasm, starch granules, *Waxy*

## Abstract

Head rice yield (HRY) is an essential quality trait, and is sensitive to environmental stresses during the grain-filling, harvest, and postharvest stages. It is therefore important for rice production and global food security to select for superior HRY traits; however, the molecular basis of this trait remains unknown. Using diverse rice germplasm material, we performed a genome-wide association study of grain fissure resistance (GFR), the phenotype most associated with HRY, and found that the granule-bound starch synthase I gene *Waxy* is an important gene controlling GFR. Analysis of near-isogenic lines demonstrated that genetic variations in *Waxy* conferred different levels of tolerance to fissuring in grains. The null allele *wx* resulted in the highest GFR, while alleles that increased amylose synthesis reduced GFR. Increases in amylose content led to increases in the ratio of the widths of the amorphous layer to the semi-crystalline layer of the starch granules, and also to increased occurrence of chalkiness. The layer structure determined GFR by affecting the degree of swelling of granules in response to moisture, and chalkiness acted as an accelerator of moisture infiltration to rapidly increase the number of swelling granules. Our study reveals the molecular basis of GFR and HRY, thus opening the door for further understanding of the molecular networks of GFR and HRY.

## Teaser

The amylose synthase gene *Waxy*affects grain fissure resistance and head rice yield by altering the layered structure of starch granules, thereby affecting their swelling response to moisture.

## Introduction

Rice (*Oryza sativa*) feeds about half of the world’s population. Head rice yield (HRY), the proportion of the milled grains retaining at least three-quarters of their original length, is an important quality trait and is considered as an index of grading. Indeed, HRY directly determines the commercial value of the grain, and head rice is priced 20–50% higher than broken rice in international trade. Reductions in HRY due to breakage during grain dehulling and milling is estimated to cause up to 30% loss of commercial yield from paddy fields (Butardo and Sreenivasulu 2019). Accelerated global warming is expected to further lower HRY, with a predicted 9–14% decrease accompanying a 1 °C elevation of temperature during grain-filling (Lyman *et al.*, 2013). Hence, HRY improvement is not only crucial for enhancing rice yield and global food security, but also for reducing economic losses and raising revenues for rice producers.

HRY is a complex trait that is determined by heredity and its interactions with environmental conditions, especially moisture and temperature, during grain-filling, harvest, drying, and storage. Although significant advances have been achieved in grain harvest and postharvest management for reducing the incidence of rice breakage (Butardo and Sreenivasulu, 2019), developing elite cultivars with superior HRY traits through molecular breeding is vital to rice production, because decreases in HRY caused by fissures occurring within damp environments is unlikely to be avoided even with careful design of machines and/or optimization of drying and tempering processes (Waggoner *et al.*, 2003). With the aim of genetically improving HRY, mapping of quantitative trait loci (QTLs) and genome-wide association studies (GWAS) have identified 52 HRY QTLs, which are distributed across all the 12 chromosomes and have been detected on the short arm of Chr 6 in at least six different mapping populations (Hao *et al.*, 2009; Bao, 2019; Misra *et al.*, 2019; Liu *et al.*, 2020). However, none of HRY QTLs have been finely mapped, mainly because the trait is extremely complex, being associated with kernel fissures as well as chalkiness and the maturity of the grains, and it is greatly affected by pre- and postharvest environments.

Formation of fissures or hairline cracks perpendicular to the longitudinal axis of brown rice prior to milling greatly reduces grain strength, and hence is the major cause of breakage and subsequent reduction in HRY; QTLs conferring grain fissure resistance (GFR) are causally connected with increases in HRY (Buggenhout *et al.*, 2013; Butardo and Sreenivasulu, 2019). Dissection of QTLs involved in GFR is a good strategy for understanding the genetic basis of HRY because the GFR phenotype can be accurately and repeatably evaluated by inducing kernel cracks under controlled environments, thereby avoiding the great phenotypic variability that is found under variable, natural conditions (Pinson *et al.*, 2012). Using this simplified system, previous studies using recombinant inbred lines derived from crosses of two high-HRY *japonica* cultivars from the USA, Cybonnet and Saber, have uncovered five GFR QTLs distributed on Chrs 1, 5, 8, and 12 (Pinson *et al.*, 2013, 2018). One locus on Chr 1 was located within a small 640-kb region (Sater *et al.*, 2017). Although genes still remain to be cloned, these studies provide a powerful approach for finding QTLs associated with HRY.

In an effort to discover QTLs for HRY, in this study we evaluated the percentages of fissured grains in 286 diverse rice germplasms using the optimized fissure-induction approach (Pinson *et al.*, 2012). Using GWAS and analysis of near-isogenic lines, we found that genetic variations in the granule-bound starch synthase I gene *Waxy* (*Wx*), which governs amylose synthesis in crops and is a well-known controller of cooking and eating quality in rice, are associated with differences in GFR. Our results suggest that *Wx*-mediated changes in the amorphous and semi-crystalline layers of the starch granules influence their swelling behaviour and hence GFR under damp conditions. In addition, chalkiness appears to act as an accelerator of moisture infiltration to increase the number of swelling starch granules.

## Materials and methods

### Plant materials

We generated and genotyped two CRISPR/Cas9-mediated *Wx*^*b*^ knockout lines (CL1 and CL2) in the background of *Oryza sativa* subsp. *japonica* Nipponbare as described by Liu *et al.* (2016). To compare *Wx* expression in Nipponbare and the two knockout lines, the GBSSI proteins were extracted and detected as described by Zhang *et al.* (2012). We also used near-isogenic lines (NILs) developed by separate introgression of the alleles *Wx*^*a*^ (from Teqing), *Wx*^*in*^ (from IR64), and *Wx*^*b*^ (from Minghui 63) into a *wx*-type *indica* rice variety (Yangfunuo 4), as described by Zhang *et al.* (2012).

All the plants of the 286 rice varieties (which included both *indica* and *japonica*; Supplementary Table S1), the two CRISPR-knockout lines, and the *Wx* NILs were grown in paddy fields amended with similar amounts of fertilizers, using standard local management practices during the summer growing seasons when the fluctuations in temperature and humidity were relatively small. The 286 rice varieties were grown at Yangzhou, Jiangsu Province, in 2016 and at Lingshui, Hainan Province, in 2017. The *Wx* NILs and the CRISPR-knockout lines were grown at Lingshui in 2018 and at Institute of Botany, Chinese Academy of Sciences, Beijing, in 2019, respectively. The varieties were planted in three batches at 15–20-d intervals according to their expected days to maturity, which ensured that they all matured around the same date (within 10 d). Plants were grown in plots of 31 × 43 m at a spacing of 20 × 15 cm. To minimize grain fissuring in the field, the plants were harvested individually once their panicles turned yellow and matured whilst still looking moist and pliable. For slow drying and minimization of grain fissuring during storage, the harvested panicles were spread on dry floors in a well-ventilated room and kept under shade at room temperature for at least 3 months to allow the grain moisture to decrease and equilibrate to ~12%. These protocols resulted in low proportions of fissured grains before the fissure-induction experiments (Supplementary Fig. S1), which was crucial to the accurate evaluation of grain fissure resistance.

### Evaluation of grain fissure resistance, head rice yield, and chalkiness

The percentage of fissured grains was evaluated under controlled laboratory conditions by an adapted fissure-induction approach based on the method of Pinson *et al.* (2012). Mature paddy rice was detached from the shade-dried panicles and evenly spread over a small nylon mesh bag before being placed into a KMF 240 constant climate chamber (Binder). To induce fissures, the rough grains in the climate chamber were first equilibrated at 22 °C and 12% relative humidity (RH) for 30 min. They were pre-treated at 46 °C and 12% RH for 60 min, then treated at 46 °C and 98% RH for 45 min, and finally dried at 22 °C and 12% RH for 24 h. The temperature and humidity deviations were less than 2 °C and 5%, respectively. After fissure induction, the grains were dehulled by hand, and plump and non-diseased brown rice was selected for examination of fissured and chalky grains. The rice of most varieties was examined using an RGQI20A grain quality inspector (Satake), which can automatically image individual grain. Some varieties had long or red grains that could not be recognized by the RGQI20A device, and therefore they were examined using a TX-200 Grainscope (Kett). The four *Wx* NILs were also examined using the Grainscope.

To assess the effects of sun-drying or drenching on HRY, newly-harvested grains were spread on an outdoor cement floor and exposed to the sun for 7 d (10 h d^–1^ in sunlight, from 8 a.m. to 6 p.m.) or to light rain for 30 min. To determine the percentages of head rice, at least 100 plump and non-diseased grains were dehulled with a JLJG 45 dehuller (Woliang) and milled with a Pearlest Polisher (Kett) for 40 s before counting the head rice.

To determine the moisture tolerance time, totally translucent grains and grains with a white-belly longer than one-third of their length were selected from the hand-dehulled, plump, and non-diseased brown rice. These grains were soaked in water at 25 °C and inspected regularly using the Grainscope to determine how long it took for the first crack to appear.

### DNA extraction, sequencing, genotyping, and genome-wide association study

The genomic DNA of the different rice genotypes was extracted from leaves from 1-week-old seedlings using a DNeasy Plant Mini Kit (Qiagen) following the manufacturer’s instruction, and then sequenced by Annoroad Gene Technology Co., Ltd (Beijing), on a HiSeq 2500 PE125 platform (Illumina) with 500-bp libraries. The raw sequencing data were processed by removal of adaptors and low-quality reads. The clean reads were mapped using BWA (v.0.7.12; http://bio-bwa.sourceforge.net/) against the reference *japonica* genome (Nipponbare, IRGSP-1 v.7.0; Kawahara *et al.*, 2013) and then processed using Samtools (v.0.1.18; https://www.htslib.org/). Single-nucleotide polymorphism (SNP) calling for each variety was performed using the HaplotypeCaller function of GATK (v.3.2-2; https://gatk.broadinstitute.org/hc/) following the best-practice workflow. SNPs were filtered by applying the following criteria: minor allele frequency (MAF) ≥0.05 and missing rate <0.5. SnpEff (http://snpeff.sourceforge.net/) was used to annotate the effects of SNPs. Principal components analysis (PCA) was conducted using the twstats program within EIGENSOFT (http://www.hsph.harvard.edu/alkes-price/software/) and linkage disequilibrium (LD) was calculated using PLINK as described by Wang *et al.* (2020). The fissured-grain percentage of the 286 varieties was used for a genome-wide association study (GWAS) using GEMMA, which uses a linear mixed model for association analysis (Zhou and Stephens, 2012). LD analysis was performed around the significant SNP of Chr 6 using Haploview as described by de Abreu Neto *et al.* (2016).

### Starch extraction

Grains milled in the Pearlest Polisher and were used to isolate starch granules by the neutral protease method (Zhang *et al.*, 2013). The isolated starch was oven-dried and stored at –20 °C before use.

### Measurement of grain dimensions and contents of amylose, proteins, and moisture

Brown rice was visually selected to remove immature and diseased grains. For most varieties, the plump and non-diseased dehulled grains were examined using the RGQI20A inspector to determine the grain length, width, and thickness. For varieties with long or red grains and the *Wx* NILs, the grain dimensions were measured using digital vernier calipers. Grain weight was measured as the weight of 100 grains using an electronic balance (Sartorius BSA-323S, 0.001 g readability) and converted to 1000-grain weight.

The dehulled grains used for the evaluation of dimensions were then examined using a Diode Array 7200 Near-Infrared Reflectance (NIR) Analyzer (PerkinElmer) to estimate the amylose, protein, and moisture contents of each variety as described by Sun *et al.* (2014). To measure the amylose content, the extracted starch was pre-treated with ethanol to remove lipids and then analysed with an Amylose/Amylopectin Assay Kit (Megazyme) according to the manufacturer’s assay procedure.

### Measurement of tensile strength

The breaking force of plump and uncracked brown rice was measured by the three-point bending test using a TA.XT *plusC* Texture Analyser (Stable Micro Systems) and used to calculate the grain tensile strength as described by Wetchakama *et al.* (2019).

### Assessment of moisture infiltration by DTAF staining

To trace the process of moisture infiltration, 5-(4,6-dichlorotriazinyl) aminofluorescein (DTAF) staining of grains and starches was performed using the method of Hsieh *et al.* (2020), with minor modifications. Briefly, brown rice was soaked in water at room temperature for either a specific time or until the first crack occurred, and then the grains were either split down the middle or along the crack line. The pieces of the grains were then incubated in triethylamine with shaking for 1 h, after which chloroform was added and they were incubated for another 30 min. A stock solution of DTAF (Sigma-Aldrich; 100 μl, 10% w/v in dimethyl sulfoxide) was then mixed in and the samples were left for 24 h in the dark, followed by washing with absolute ethanol three times at 15-min intervals. The washed pieces of the grains were then dried in a Savant ISS110 SpeedVac (Thermo Scientific) for 30 min and stored at 4 °C in the dark prior to imaging of green fluorescence under an Axio Imager A1 microscope (Zeiss). The same procedure was used to stain dry and drenched starch granules except that the starch was collected by centrifugation at 3000 *g* for 10 min in transfer steps. Stained starch granules were imaged under an FV1000MPE laser confocal microscope (Olympus).

The moisture infiltration depth of an individual starch granule was considered as the longest distance from the bright fluorescent boundary that appeared in the endosperm after water soaking to the grain edges on the ventral, lateral, and dorsal sides, as measured using the Image-Pro Plus (IPP) software (https://www.mediacy.com/).

### Dynamic vapor sorption

To measure changes in grain mass in response to high humidity, three translucent or white-belly dehulled grains were placed into a Dynamic Vapor Sorption (DVS) Advantage-1 instrument (Surface Measurement Systems). The temperature was held at 25 °C. The relative humidity was 0% for the first 10 min and then directly increased to 98%, at which point changes in mass began to be recorded at 15-min intervals. Samples of extracted starch (3 mg) were evenly spread on the pan of the DVS instrument and subjected to the same procedure to obtain weight increase data. The percentage increase in weight was calculated at each time-point relative to the original weight at 0% humidity.

### High-speed microscopy imaging

For sequential image capture of the occurrence of fissures, a translucent or white-belly grain was selected, soaked in water, and inspected using the Grainscope. An S8APO stereomicroscope (Leica) equipped with a pco.dimax cs3 high-speed camera (PCO, Kelheim, Germany) was used to image the occurrence of fissures at a speed of 2000 frames s^–1^.

### Quantitative assessment of starch swelling in water

The extracted starches were treated with 8% HCl at 38 °C for 2 d and then washed with water three times. After drying at 40 °C for 48 h, these samples together with untreated, dry samples were dispersed evenly over double-sided tape attached to microscope slides and imaged under the Axio Imager A1 microscope. Water at room temperature was added, and soaked starch granules at the same position were imaged again. The IPP software was used to evaluate the degree of swelling of each individual starch granule on the basis of its area.

### Scanning electron microscopy

To observe the natural fracture surfaces of fissured grains, broken brown rice induced by the high-humidity (98%) treatment described above was used directly as samples. For observation of starch granules on a cross-section of endosperm, brown rice was frozen in liquid nitrogen for 5 min and broken in half using tweezers. For examination of starch growth rings, extracted starch granules were cracked and hydrolysed with α-amylase for 2 h according to the method described by Pilling and Smith (2003). The prepared samples were mounted on specimen stubs, sputter-coated with gold, and observed under an S-4800 Field Emission SEM (Hitachi High-Tech). The IPP software was used to measure the width of starch growth rings.

### X-ray powder diffraction

X-ray diffractograms were collected using an Empyrean diffractometer (Malvern Panalytical, UK) at 30 kV and 30 mA. The scanning 2θ angles ranged from 3.5° to 50°, and the scan speed was 1° min^–1^. The relative crystallinity of the starch granules was quantitatively calculated by separating the diffractograms into the crystalline and amorphous portions as described by Nara and Komiya (1983).

### Fourier-transform infrared spectroscopy

Fourier-transform infrared spectroscopy (FTIR) spectra of the extracted starch were recorded on a Tensor 27 spectrometer (Bruker) equipped with a deuterated triglycine sulfate detector using an attenuated total reflectance accessory at a resolution of 4 cm^–1^. Spectra were baseline-corrected and deconvoluted in the wavenumber region from 1200 cm^–1^ to 800 cm^–1^ using Bruker Opus 3.0. Infrared absorbance values at 1047 cm^–1^ and 1022 cm^-1^ were extracted from the baseline-corrected and deconvoluted spectra to assess the short-range ordered structure of the starch.

### Size-exclusion chromatography

The extracted starch was dissolved in DMSO solution containing LiBr (0.5% w/w), debranched by isoamylase, then subjected to size-exclusion chromatography (SEC) using an LC-20AD SEC system coupled with an RID-10A refractive index detector (both Shimadzu) as described by Zhong *et al.* (2020). The flow rate was 0.6 ml min^–1^ and a combination of GRAM100 and GM1000 analytical columns (PSS Polymer Standards Service) was used to analyse the chain-length distribution of the debranched starch molecules.

## Results

### Allelic variations in *Wx* are associated with grain fissure resistance and head rice yield

**Fig. 1.**
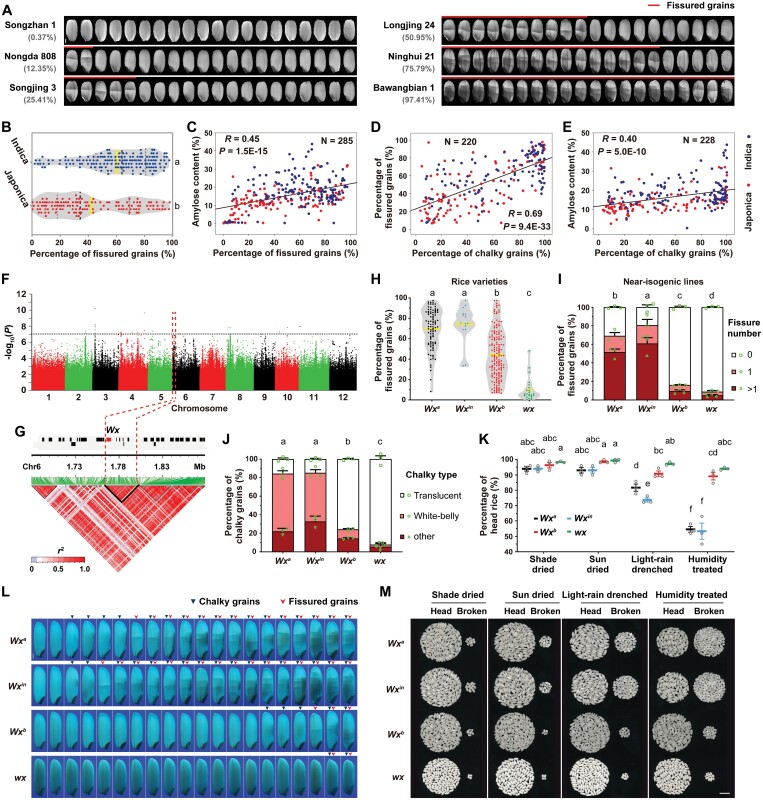
*Waxy* affects grain fissure resistance and head rice yield. (A) Representative grain images showing examples of rice varieties with different percentages of fissured grains after high-humidity induction [equilibrated at 22 °C and 12 % relative humidity (RH) for 30 min, then treated at 46 °C and 12 % RH for 60 min followed by 98% RH for 45 min, and finally dried at 22 °C and 12 % RH for 24 h]. The variety names and percentages are indicated. The scale bar is 1 mm. (B) Distribution of fissured-grain percentages in *japonica* (*n*=112) and *indica* (*n*=174) varieties. Yellow indicates the means, the black dashed lines indicate the medians, and the gray dashed lines indicate the first and third quartiles. Each dot represents the mean value of two independent evaluations of one variety. (C–E) Correlations between (C) fissured-grain percentage and amylose content, (D) chalky-grain percentage and fissured-grain percentage, and (E) chalky-grain percentage and amylose content. The Pearson correlation coefficients (*R*) and associated *P*-values are shown. N indicates the number of rice varieties used for analysis. (F) Manhattan plot of the results of a genome-wide association study on fissured-grain percentages of the 286 rice varieties (Supplementary Table S1). (G) Linkage disequilibrium (LD) structure around the associated locus in chromosome 6. The rectangles represent genes located within this locus, with *Waxy* (*Wx*) highlighted. The color key shows the LD values (*r*^2^). LD blocks are outlined in black. The red dashed lines mark the boundaries of the LD block containing the peak SNP of the region. (H) Distribution of fissured-grain percentages in 258 rice varieties carrying either the *Wx*^*a*^ (*n*=89), *Wx*^*in*^ (*n*=15), *Wx*^*b*^ (*n*=131), and *wx* (*n*=23) alleles (Supplementary Table S1). Each dot represents the mean value of two independent evaluations of one variety. (I) Fissured-grain percentages of four *Wx* near-isogenic lines (NILs) following the high-humidity treatment. Data are means (±SE) of three experiments, with at least 100 high-humidity treated grains for each experiment. (J) Chalky-grain percentages of the four *Wx* NILs. Data are means (±SE) of the same samples in (I). (K) Head-rice percentages of the four *Wx* NILs assessed after different grain-drying and moisture treatments. Newly-harvested grains were shade-dried at room temperature for at least 3 months, sun-dried for 7 d, drenched with light-rain for 30 min, or treated at 98% RH humidity for 45 min before dehulling and milling for counting head rice. Data are means (±SE) of three assessments, with at least 100 newly harvested grains for each test. (L) Representative images of grains of the four *Wx* NILs following the high-humidity treatment showing differences in the proportions of fissured and chalky grains. The scale bar is 1 mm. (M) Representative images of head and broken rice after different grain-drying and moisture treatments, as described in (K). A total of 100 dried or moisture-treated grains were dehulled and milled. The scale bar is 1 cm. In (B), (H), (I), (J) and (K), different letters indicate significant differences among means as determined using one-way ANOVA followed by Tukey’s test (*P*<0.01).

To explore the correlative factors affecting variations in GFR within rice, we collected 286 diverse germplasms comprising 112 *japonica* and 174 *indica* varieties, both of which had rapid linkage disequilibrium (LD) decay (Supplementary Table S1, Supplementary Fig. S2), and evaluated their percentage of fissured grains under controlled laboratory conditions using a fissure-induction approach. The results were highly variable across the different varieties, ranging from 0.34% to 97.69%, and the mean value was <45% for the *japonica* varieties whereas it was ~60% for the *indica* varieties (Fig. 1A, B). The amylose content of mature dry grains was significantly positively correlated with the percentage of fissured grains (Fig. 1C) whilst no significant correlations were observed for the protein and moisture contents (Supplementary Fig. S3A, B). The percentage of chalky grains was also significantly positively correlated with the percentage of fissured grains, and there was a positive correlation between the amylose content and the percentage of chalky grains (Fig. 1D, E). The length, width, thickness, and length-to-width ratio of grains did not appear to be connected with the percentage of fissured grains (Supplementary Fig. S3C–F).

Next, we detected QTLs for GFR through a GWAS of the fissured-grain percentage among the 286 rice varieties using a mixed linear model. We identified three significantly associated loci on Chrs 3, 6, and 8 (Fig. 1F; Supplementary Fig. S4A). Only the associated locus on Chr 6, which we termed *FISSURE RESISTANCE DIVERGENCE 1* (*FED1*), was detected in both years of our experiments, which might have been the result of the minor variations between the data for fissured-grain percentage in the two years (Supplementary Fig. S4B–E). The peak SNP of the *FED1* region was located in a 36-kb LD block spanning six genes: *LOC_Os06g04200*, *LOC_Os06g04210*, *LOC_Os06g04220*, *LOC_Os06g04230*, *LOC_Os06g04240*, and *LOC_Os06g04250* (Fig. 1G). The peak SNP (Chr 6: 1 779 126, *P*=1.70 × 10^–10^) fell in the intergenic region between the functionally unknown *LOC_Os06g04210* and *LOC_Os06g04220*. In addition to the peak SNP, only one SNP (Chr 6: 1 765 761, *P*=1.07 × 10^-9^) in the 36-kb LD block had a *P-*value less than the suggestive threshold (*P*<1 × 10^–7^), and it was located in the splice donor site of the first intron of *LOC_Os06g04200*. This is the well-known rice granule-bound starch synthase I (GBSSI) gene *Waxy* (*Wx*), which governs amylose synthesis in the endosperm. Considering the positive correlation between the amylose content and the fissured-grain percentage, *Wx* is the most likely candidate gene underlying the *FED1* locus.

There were four main *Wx* alleles among the 286 rice varieties, namely *Wx*^*a*^, *Wx*^*in*^, *Wx*^*b*^, and *wx*. Compared to *Wx*^*a*^, *Wx*^*in*^ had a missense mutation of adenine (A) to cytosine (C) in the sixth exon, resulting in the substitution of serine for tyrosine (Supplementary Fig. S4F, G) and a consequent reduction in GBSSI enzyme activity (Zhang *et al.*, 2012). The guanine (G) to thymine (T) splicing mutation on the second significant SNP site detected by GWAS was present in both *Wx*^*b*^ and *wx* (Supplementary Fig. S4F, G) and causes a large decrease in *Wx* mRNA expression (Hirano *et al.*, 1998; Isshiki *et al.*, 1998). On top of the splicing mutation, the *wx* allele contained a 23-bp insertion in exon 2 (Supplementary Fig. S4F, G), leading to the almost complete absence of GBSSI (Sano, 1984). The varieties were divided into four groups according to their *Wx* genotypes (258 varieties in total). The mean percentage of fissured grains was lowest in the *wx* group (known as glutinous rice), and was significantly less in the *Wx*^*b*^ group compared with the *Wx*^*a*^ and *Wx*^*in*^ groups (Fig. 1H). Most of the *japonica* cultivars (74/103) carried the *Wx*^*b*^ allele while more than half of the *indica* varieties (88/155) carried the *Wx*^*a*^ allele (Supplementary Table S1), which accounts for the relatively lower percentages of fissured grains in *japonica* (Fig. 1B).

We further confirmed the association of these *Wx* allelic variations with GFR using NILs developed by separate introgression of *Wx*^*a*^, *Wx*^*in*^, and *Wx*^*b*^ into a *wx*-type *indica* rice variety (Zhang *et al.*, 2012). The four lines showed little difference in yield traits, except that grains of NIL-*wx* and NIL-*Wx*^*b*^ were slightly smaller than those of NIL-*Wx*^*a*^ and NIL-*Wx*^*in*^ (Supplementary Fig. S5). There were hardly any fissured grains when the four NILs were just harvested and shade-dried, but after high-humidity treatment, relatively high levels were observed in NIL-*Wx*^*in*^ and also in NIL-*Wx*^*a*^ (Fig. 1I, L). In comparison, NIL-*Wx*^*b*^ had relatively small fractions of fissured grains and NIL-*wx* showed the lowest percentage, indicating that these two NILs were highly resistant to grain fissuring. Taken together with the GWAS, these results indicated that *Wx* is an important factor in relation to variations in GFR. In addition, NIL-*Wx*^*a*^ and NIL-*Wx*^*in*^ had much higher percentages of chalky grains than NIL-*Wx*^*b*^ and NIL-*wx* (Fig. 1J, L). Newly harvested grains that were either shade-dried or sun-dried both had a high percentage of head rice, although there were subtle differences among the four NILs (Fig. 1K, M). However, the head-rice percentages of NIL-*Wx*^*a*^ and NIL-*Wx*^*in*^ became much lower than those of NIL-*Wx*^*b*^ and NIL-*wx* after dried grains were exposed to damp conditions, both when drenched with light-rain or treated with high humidity. These results showed that genetic variations in *Wx* mediated GFR, chalkiness, and HRY.

To further verify the role of *Wx*, we also used the CRISPR/Cas9 system to knockout *Wx* in the *japonica* cultivar Nipponbare carrying the *Wx*^*b*^ allele and obtained two Cas9-free *Wx*^*b*^-knockout lines (Supplementary Fig. S6A–C). Compared to the wild-type, both the knockout lines (T3 generation) showed a significantly decreased percentage of fissured grains and an increased percentage of head rice after high-humidity treatment (Supplementary Fig. S6D, F–H). These results provided further evidence that *Wx* affects GFR and HRY. In addition, the mutations in *Wx*^*b*^ led to reductions in chalkiness and grain size (Supplementary Fig. S6E, F, I–M).

We next investigated the effects of grain fissuring and chalkiness on HRY by dividing grains of each NIL into translucent and white-belly groups and examining whether they were different in their percentage of head rice. In both groups after grain milling, practically all unfissured grains were intact while more than half of fissured grains were broken (Supplementary Fig. S7), suggesting that grain fissuring rather than chalkiness contributed directly to HRY reduction.

### Translucent and chalky grains show differences in GFR

**Fig. 2.**
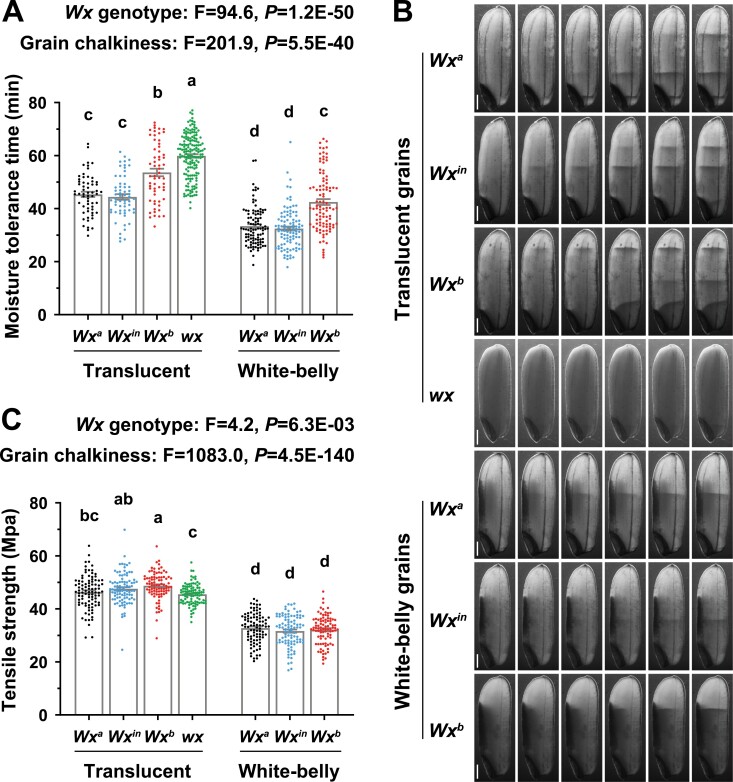
Grain fissure resistance of translucent and chalky grains from the four rice *Waxy* (*Wx*) near-isogenic lines (NILs). (A) Moisture tolerance time of translucent and white-belly grains. Hand-dehulled, plump, and non-diseased brown rice was soaked in water at 25 °C and the time taken for the first crack to appear was recorded. Data are means (±SE), *n*≥60 grains. (B) High-speed sequential imaging of grains showing the initiation and development of fissures in translucent and white-belly grains of the NILs. Scale bars are 1 mm. (C) Tensile strength of translucent and white-belly grains. The breaking force of plump and uncracked brown rice was measured using a three-point bending test. Data are means (±SE), *n*≥82 grains. Different letters indicate significant differences among means as determined using one-way ANOVA followed by Tukey’s test (*P*<0.01). The *F*- and *P* values were generated by multi-factor variance analyses.

Because the percentage of chalky grains was positively correlated with the percentage of fissured grains (Fig. 1D), we examined whether the translucent and white-belly groups differed in their moisture tolerance durations. On average, cracks appeared much sooner in the white-belly grains than in the translucent ones from the same NIL (Fig. 2A), indicating that chalkiness had an effect on GFR. When comparing the four NILs within each of the translucent and white-belly groups, the grains of NIL-*Wx*^*a*^ and NIL-*Wx*^*in*^ on average developed fissures more rapidly than those of NIL-*Wx*^*b*^ and NIL-*wx*, suggesting another aspect was involved in *Wx*-mediated GFR besides its influence through chalkiness.

High-speed stereomicroscopy showed that fissure initiation and development were clearly different between the translucent and white-belly grains. In translucent kernels regardless of the *Wx* genotype, every fracture appeared initially in the middle and then through to the periphery of the endosperm (Fig. 2B; Supplementary Movie S1). By contrast, white-belly grains of the NILs cracked from the chalky ventral portion to the dorsal side, although in all these cases fissures were developed along the transverse direction perpendicular to the longitudinal axis of the grains. These observations confirmed the involvement of two different aspects in *Wx*-mediated GFR.

Grain fissures are presumed to develop when the tensile strength of grains is not great enough to withstand the internal stresses caused by moisture diffusivity (Buggenhout *et al.*, 2013), so we next compared the tensile strengths of the translucent and white-belly grains of the *Wx* NILs. Whilst only subtle differences among the results from the translucent grains, white-belly grains were generally much weaker than the translucent ones (Fig. 2C). The fact that tensile strength was therefore closely connected with chalkiness rather than with the *Wx* genotype suggested that the weakness of white-belly grains might partly account for their low tolerance to moisture treatment, but tensile strength was unrelated to the differences in GFR among the different *Wx* NILs within the translucent and white-belly groups.

Grain fissures occur in relatively vulnerable parts of the endosperm, and we next examined whether fissured grains of the four *Wx* NILs showed differences in their natural fracture surfaces. SEM observations demonstrated that the grain fracture surfaces of the NILs were similar in morphology (Supplementary Fig. S8A). The fracture surfaces of all the broken grains, whether translucent or chalky, were decorated with randomly distributed smooth endosperm cell interfaces and rough granular surfaces filled with exposed individual or compound starch granules (Fig. S8A, B). These results indicated that the grain fracture surfaces had no connection with the variations in GFR.

### *Wx* influences chalkiness, which accelerates moisture infiltration in grains

It has been assumed that moisture transfer and the resultant moisture gradients within a rice grain raise the mechanical stress inside its endosperm, thus inducing transverse cracks within it (Buggenhout *et al.*, 2013). However, there is no solid evidence for this hypothesis, because there are no imaging tools that are able to directly examine moisture diffusion within grains. Here, we used 5-(4,6-dichlorotriazinyl) aminofluorescein (DTAF), a fluorescent dye applied to study starch hydration and infiltration of reagents (Hong *et al.*, 2016; Hsieh *et al.*, 2020), to trace the process of moisture infiltration into whole grains during treatment.

**Fig. 3.**
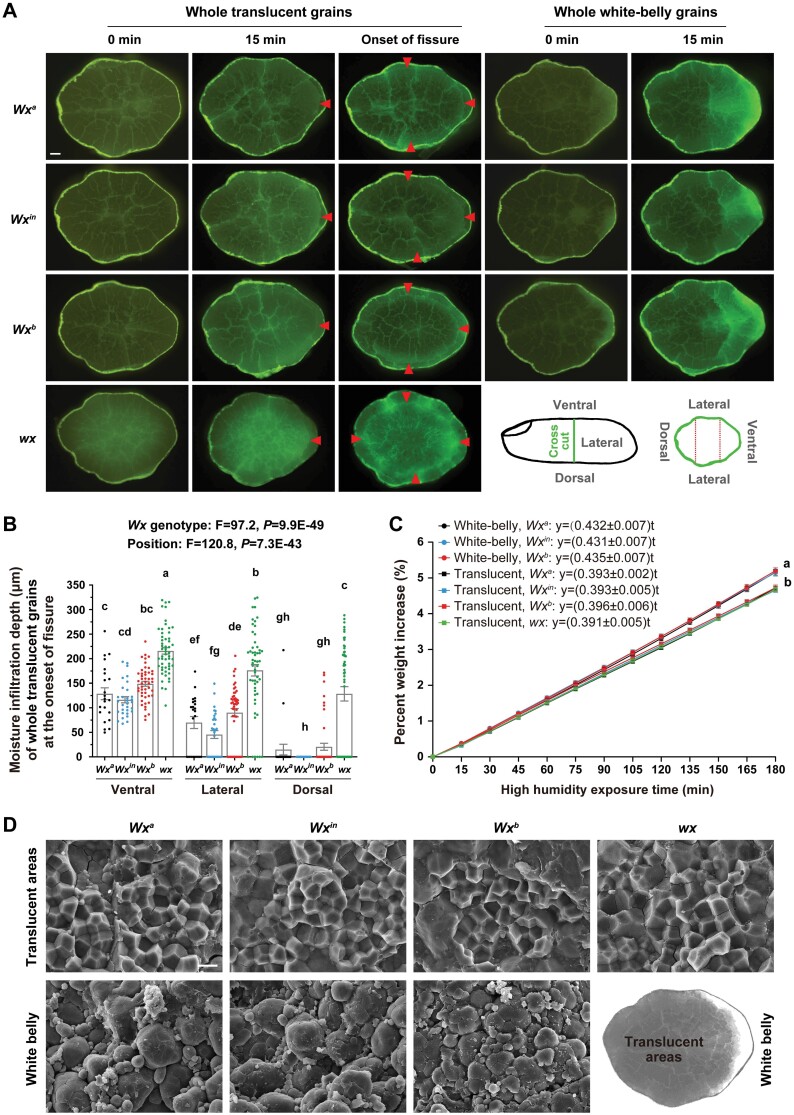
Chalkiness associated with *Waxy* (*Wx*) in the four rice near-isogenic lines affects moisture infiltration in grains. (A) Representative images of cross-sections of DTAF-stained translucent and white-belly grains after soaking in water for 0 min and 15 min, and at the time when fissuring was first observed (Onset of fissure; see Fig. 2A, B). Red arrowheads mark the bright boundaries showing the depth of moisture infiltration. The scale bar is 200 μm. The positions of the different parts of the grain are shown in the diagram. (B) Moisture infiltration depth of whole translucent grains in the different grain parts at the onset of fissure. Data are means (±SE), *n*=12–22 grains. Different letters indicate significant differences among means as determined using one-way ANOVA followed by Tukey’s test (*P*<0.01). The *F*- and *P*-values were generated by multi-factor variance analyses. (C) Percent weight increases of translucent and white-belly grains after different lengths of exposure to high humidity (98%), as determined by dynamic vapor sorption analysis. The moisture absorption rates calculated from the graphs are indicated. Data are means (±SE) of four analyses, with three grains for each analysis. The different letters indicate significant differences among means as determined using one-way ANOVA followed by Tukey’s test (*P*<0.01). (D) The structure of translucent and white-belly endosperm observed by SEM. The diagram shows the translucent and white-belly areas. The scale bar is 5 μm.

Dry translucent grains could not be DTAF-stained and only showed background fluorescence (Fig. 3A). After soaking for 15 min, a bright boundary appeared on the ventral side of the endosperm of translucent grains, indicating the depth of moisture infiltration (Fig. 3A). Thereafter, the bright boundary moved steadily towards the inner areas, in addition to expansion to the lateral side and then to the dorsal side of the endosperm (Supplementary Fig. S9A). At 5 h of treatment, moisture had dispersed to nearly the whole endosperm (Supplementary Fig. S9A). Multi-factor variance analysis showed that the moisture infiltration depth in the translucent grains was generally independent of the *Wx* genotype and chiefly dependent on soaking time (Supplementary Fig. S9B). In addition, we compared the moisture infiltration depth at the onset of fissuring and found that the translucent grains of NIL-*Wx*^*b*^ and NIL-*wx* needed wider diffusion of water to triggering fissuring compared with those of NIL-*Wx*^*a*^ and NIL-*Wx*^*i*n^ (Fig. 3A, B), which was consistent with their moisture tolerance times (Fig. 2A).

For the white-belly kernels, it only took 15 min of soaking to result in very strong green fluorescence around the chalky ventral regions, similar to that observed after 5 h in the translucent grains (Fig. 3A; Supplementary Fig. S9A). This demonstrated the accelerating effect of chalkiness on moisture infiltration in the grains.

We next quantified the moisture absorption rate of the grains using dynamic vapor sorption (DVS) analysis. On exposure to high humidity, kernels steadily absorbed moisture and their weight increased linearly with the time of exposure (Fig. 3C). The moisture absorption rate was significantly higher in white-belly grains than in translucent ones regardless of the *Wx* genotype. Thus, both the DTAF and DVS results suggested that chalkiness accelerated moisture absorption in grains, and this could be explained by the structural differences that we observed between the endosperms of the translucent and chalky grains. Polyhedral starch granules were densely packed in the translucent-grain endosperm while irregular, rounded granules were loosely packed in white-belly endosperm (Fig. 3D). The interstices and cavities in the chalky endosperm seemed to link together to form capillary-like networks that would facilitate moisture diffusivity.

### *Wx* NILs display variations in the degree of starch swelling

According to the principles of material mechanics, hygroscopic swelling induces mechanical stresses inside structures, and therefore we examined whether the variation in GFR among the four *Wx* NILs resulted from their differences in hygroscopic behavior and the consequent differences in the swollen state of their starch. Comparison of the moisture absorption behaviors of the starch granules in response to high humidity detected by DTAF-staining among the NILs indicated that dry starch granules showed relatively dim fluorescence on their exteriors while wet granules were entirely brilliant green (Supplementary Fig. S10A), demonstrating that the granules from all the *Wx* NILs are able to thoroughly absorb water. Further DVS examination of the moisture sorption behaviors of the thin layers of starch granules indicated that there were no statistically significant differences among the *Wx*^*a*^, *Wx*^*in*^, and *Wx*^*b*^ alleles (Supplementary Fig. S10B).

**Fig. 4.**
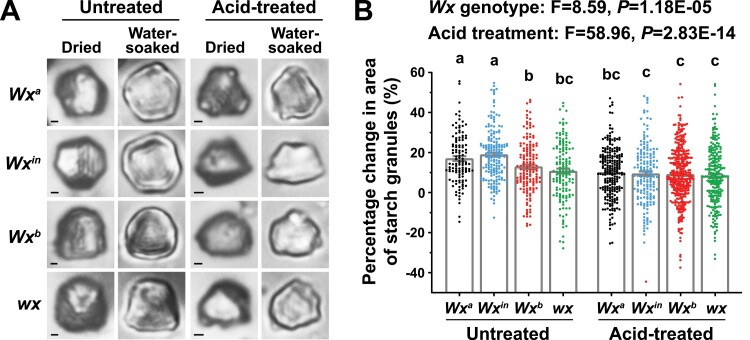
*Waxy* (*Wx*) influences the degree of starch swelling in rice grains. Starch granules in the *Wx* near-isogenic lines were extracted using the neutral protease method. For acid treatment, extracted granules were treated with 8% HCl at 38 °C for 2 d, and then dried at 40 °C for 48 h before being soaked in water at room temperature. The acid treatment results in a great part of the starch amorphous regions being hydrolysed while the semi-crystalline regions are preserved. (A) Representative images of granules with and without acid treatment before and after water soaking. Scale bars are 5 μm. (B) Percentage changes in the areas of starch granules after soaking in water with or without acid treatment. Data are means (±SE), *n*≥117 granules. Different letters indicate significant differences among means as determined using one-way ANOVA followed by Tukey’s test (*P*<0.01). Multi-factor variance analyses were used to generate the *F*- and *P*-values.

We then examined whether the starch granules in the four *Wx* NILs were different in their individual degree of expansion under a moisture-adsorbing environment. Granules swelled immediately after soaking in water, and the increase in granule size of NIL-*Wx*^*in*^ and NIL-*Wx*^*a*^ was larger on average than that of NIL-*Wx*^*b*^ and NIL-*wx* (Fig. 4). Since starch granules are semi-crystalline materials comprising alternating amorphous and semi-crystalline growth rings (Bao, 2019), we treated granules with hydrochloric acid for 2 d, resulting in a great part of the amorphous regions being hydrolysed while the semi-crystalline regions were preserved (Jayakody and Hoover, 2002). Acid hydrolysis led to a larger reduction of granule swelling in NIL-*Wx*^*in*^ and NIL-*Wx*^*a*^ than in NIL-*Wx*^*b*^ and NIL-*wx*, and consequently acid-treated granules from all the *Wx* NILs swelled to a similar extent when soaked in water (Fig. 4). These results indicated the expansion of starch granules in water was related to their amorphous regions, and variations in the degree of starch swelling among the *Wx* NILs accounted for their differences in tolerance to moisture treatment.

### The layered structure of starch granules determines their degree of swelling

**Fig. 5.**
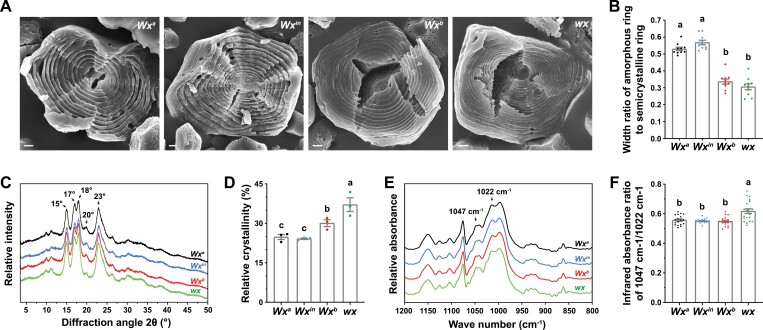
*Waxy* (*Wx*) influences the layered structure of starch in rice grains. (A) Representative SEM images of the growth rings of starch granules in the four near-isogenic lines after hydrolysis by α-amylase for 2 h, which removes the amorphous regions. The protruding rings are semi-crystalline growth rings, while the grooved rings between them are amorphous growth rings. The scale bars are 1 μm. (B) The width ratios of the amorphous growth rings to the semi-crystalline growth rings. Data are means (±SE), *n*=10 granules. (C) X-ray diffractograms of the starch granules. Rice starches show A-type patterns with prominent diffraction peaks at around 15°, 17°, 18°, 20°, and 23°, indicated by arrows on the diffractogram of *Wx*^*a*^. D. Relative crystallinity of starch granules calculated from the X-ray diffractograms. Data are means (±SE) of three experiments. (E) Fourier-transform infrared spectroscopy (FTIR) of the starch granules. The absorbance peaks at the 1047 cm^–1^ and 1022 cm^–1^ wave numbers (shown by arrows) are related to the crystalline and amorphous domains of the granules, respectively. (F) Infrared absorbance ratio of 1047 cm^–1^ to 1022 cm^–1^ extracted from the FTIR curves. Data are means (±SE), *n*=18–20 FTIR analyses. Different letters in the graphs indicate significant differences among means as determined using one-way ANOVA followed by Tukey’s test (*P*<0.01).

To clarify the differences among the four *Wx* NILs in the structure of their starch granules, we first cracked and hydrolysed granules to examine the structures of their growth rings by SEM. After hydrolysis for 2 h, protruding semi-crystalline growth rings alternating with grooved amorphous growth rings were clearly visible on the inner surfaces of center-cracked starch granules in all the four NILs (Fig. 5A). The granules of NIL-*Wx*^*in*^ and NIL-*Wx*^*a*^ showed significantly higher ratios of the width of amorphous growth rings to semi-crystalline growth rings than the NIL-*Wx*^*b*^ and NIL-*wx* lines (Fig. 5B).

The amorphous growth rings of starch granules chiefly include amylose and relatively unordered amylopectin, while the semi-crystalline growth rings consist of crystalline and amorphous lamellae repeats formed by packed helical clusters and branched segments of amylopectin, respectively (Bao, 2019). We therefore examined the long-range ordered crystallization of amylopectin double-helices by X-ray diffraction, and found that the calculated relative crystallinity of NIL-*Wx*^*b*^ starch granules was lower than that of NIL-*wx* granules but higher than that of NIL-*Wx*^*in*^ and NIL-*Wx*^*a*^ granules (Fig. 5C, D). Fourier-transform infrared spectroscopy was used to analyse the short-range double-helical structure of starches, with the infrared absorbance ratio of 1047 cm^–1^ to 1022 cm^–1^ wave-numbers reflecting the amounts of crystalline domains relative to amorphous ones (Fig. 5E). Analysis of the data demonstrated that the starch granules of NIL-*Wx*^*a*^, NIL-*Wx*^*in*^, and NIL-*Wx*^*b*^ exhibited very similar ratios and they were lower than the ratio in NIL-*wx* granules (Fig. 5F). Thus, our conformational comparison of starches from the *Wx* NILs suggested that GFR was associated with long-range crystalline order rather than short-range double-helical order.

We further examined whether the variations in starch structure among the *Wx* NILs were associated with the differences in their amylose content. Quantification of amylose using the lectin-binding method showed that NIL-*Wx*^*in*^ had the highest content followed in order by NIL-*Wx*^*a*^, NIL-*Wx*^*b*^, and NIL-*wx* (Supplementary Fig. S11A). Size-exclusion chromatography (SEC) revealed that the molecular-weight distribution of debranched starches displayed two large peaks of amylopectin chains and two small peaks of amylose chains (Supplementary Fig. S11B), representing short (AP1) and long (AP2) amylopectin chains, short amylose chains together with extra-long amylopectin branch chains (AM1) and long amylose chains (AM2), respectively. The SEC curves of the four NILs roughly overlapped at the AP1 and AP2 peaks but were clearly separated at the AM1 and AM2 peaks (Supplementary Fig. S11B), suggesting there are major differences in the chain-length distribution of amylose.

The area proportions of the AM1 together with the AM2 peaks relative to the total area of all four peaks [AM/(AM+AP)] generally corresponded to the amylose contents as determined by the lectin-binding method, with the exception of NIL-*Wx*^*in*^ (Supplementary Fig. S11C). The *Wx*^*in*^ allele carries the substitution of serine for tyrosine at the 224th residue of GBSSI, which is the key active site responsible for the biosynthesis of extra-long amylopectin chains (Hanashiro *et al.*, 2008). The AM1 section contains extra-long amylopectin chains with molecular weights similar to short amylose chains; this explains why the calculated area proportion of AM for NIL-*Wx*^*in*^ was more consistent with the apparent amylose content previously estimated by the colorimetric iodine test (Zhang *et al.*, 2012) than with the amylose content measured by the lectin-binding method. The area ratio of the AM2 section to the total AM section expresses the relative amounts of long amylose chains and it was positively related to the amylose content (Supplementary Fig. S11C). Taken together, these results indicated that the amylose content affected the crystalline order of starches, which in turn influenced their growth-ring structure, particularly that of the amorphous region, and consequently it determined the degree of starch swelling in response to moisture.

## Discussion

Head rice yield (HRY) is a crucial quality trait in the milling recovery of paddy rice, and hence it is an important target for breeders in developing elite rice varieties; however, the genetic basis underlying this trait is almost unknown. Using a genome-wide association study (GWAS) and analysis of near-isogenic lines (NILs), our study has highlighted an important gene that mediates grain fissure resistance (GFR) and HRY. Unexpectedly, this gene was identified as *Waxy* (*Wx*), which is well-known for its vital role in amylose synthesis in the endosperm of cereal crops including rice, and which is central to determining the cooking and eating quality of rice (Zhou *et al.*, 2021). Our results demonstrate that allelic variations of *Wx* are associated with differences in GFR and HRY, and that *Wx*^*b*^ confers greater tolerance to grain fissuring and higher HRY in comparison to *Wx*^*a*^ and *Wx*^*in*^, indicating that finely tuned *Wx* expression is crucial for both these traits. Hence, *Wx* can be regarded as a master module for breeding elite rice varieties, and refined editing of *Wx* at multiple levels has great potential for improving crop production.

Our study has uncovered the molecular mechanism of *Wx*-mediated GFR. We found that *Wx* allelic variations resulted in differences in the amorphous and semi-crystalline layered structure of starch granules (Fig. 5) as a result of modulation of the amylose content (Supplementary Fig. S11), which in turn determined the degree of swelling of the granules after moisture absorption (Fig. 4), consequently affecting GFR and HRY (Fig. 1I, K–M). Our results showed that the overwhelming majority of chalky kernels were robust enough to preserve their integrity during milling (Fig. 2C), and hence unfissured chalky and translucent grains gave rise to similar HRY (Supplementary Fig. S7), which disagrees with the widespread view that the negative correlation between chalkiness and HRY is due largely to the weakness of chalky kernels (Bao, 2019). We found that the reduction in HRY caused by chalkiness resulted from its increased susceptibility to fissuring, which in turn led to breakage. When a translucent kernel was exposed to damp environments, moisture permeated slowly into the endosperm in all directions (Supplementary Fig. S9), causing the expansion of soaked starch granules in the outer layers and a resultant outward stress in the middle of the endosperm. As moisture penetrated deeper into the endosperm, a growing number of granules in the outer layers became saturated and bigger, increasing the outward stress until it exceeded the strength limit of the grain and induced fissuring from the centre to the periphery of the endosperm (Fig. 2B; Supplementary Movie S1). In white-belly grain, moisture diffused rapidly throughout the chalky area due to interstices among loosely packed starch granules (Fig. 3A, C–D), so that a large number of starch granules expanded to create considerable stress in the ventral side of the grain in a short time. The stress in the ventral endosperm caused the grain to develop cracks at a high speed from the chalky belly to the back (Fig. 2B; Supplementary Movie S1). Thus, the accelerator effect of chalkiness on moisture infiltration in these grains makes fissuring more likely in adverse moisture and temperature environments than in translucent grains.

In conclusion, our study clearly elucidates the molecular mechanisms underlying GFR and HRY, and demonstrates that the amorphous and semi-crystalline layered structure of starch granules is a key structural basis for GFR, with chalkiness accelerating the degree of starch granule swelling under damp conditions. Our results open the door for further understanding of the molecular networks that determine GFR and HRY.

## Supplementary data

The following supplementary data are available at *JXB* online.

Fig. S1. Percentage of fissured grains before and after high-humidity induction of fissuring.

Fig. S2. Principal component analysis and linkage disequilibrium decay between the *japonica* and *indica* varieties.

Fig. S3. Correlations among fissured-grain percentage and protein content, moisture content, and the thickness, length, width, and length-to-width ratio of grains.

Fig. S4. Summary data for the GWAS on grain fissure resistance.

Fig. S5. Plant and grain phenotypes of the different *Wx* allelic lines.

Fig. S6. Effects of CRISPR/Cas9-mediated knockout of *Wx*^*b*^ on GFR and HRY.

Fig. S7. Head rice yield is reduced by grain fissuring rather than chalkiness in the four *Wx* NILs.

Fig. S8. SEM images of fracture surfaces of fissured grains from the four *Wx* NILs.

Fig. S9. Moisture infiltration behaviors of translucent grains of the four *Wx* NILs.

Fig. S10. Moisture absorption behaviors of starch granules of the four *Wx* NILs.

Fig. S11. Contents of amylose and amylopectin in the four *Wx* NILs.

Table S1. Details of the 286 rice varieties used in this study.

Movie S1. Time-lapse images of the initiation and development of fissures in translucent and white-belly grains from the four *Wx* NILs.

erac330_suppl_Supplementary_Figures_S1-S11

erac330_suppl_Supplementary_Table_S1

erac330_suppl_Supplementary_Movie_S1

## Data Availability

The data supporting the findings of this study are available within the paper and its supplementary data published online.
