# Supplementary material for: Waxy is an important factor for grain fissure resistance and head rice yield as revealed by a genome-wide association study
Source: J Exp Bot. 2022 Sep 2;73(19):6942–54. doi: 10.1093/jxb/erac330 (PMC9629786; doi:10.1093/jxb/erac330)
Supplement: erac330_suppl_Supplementary_Figures_S1-S11 [file erac330_suppl_supplementary_figures_s1-s11.pdf]

Supplementary Figures

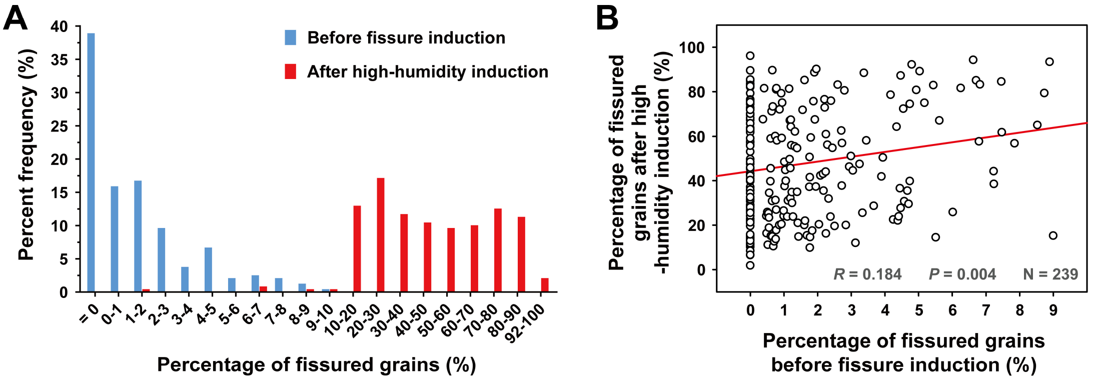

Fig. S1 Percentage of fissured grains before fissure induction is much lower than that after high-humidity induction

A. Percent frequency distribution of fissured-grain percentage before fissure induction compared with that after high-humidity induction.

B. Scatter plot of fissured-grain percentages before fissure induction and after high-humidity induction. Pearson correlation was used to generate *R* and *P* values and N indicates the number of rice varieties used in the correlation analysis.

Minimizing grain fissuring before the fissure-induction experiments is crucial to the accurate evaluation of grain fissure resistance. As we described in “Plant materials”, we used several preventative measures to try to avoid adverse pre- and post-harvest conditions and minimize grain fissuring. Here, we assessed the effectiveness of our protocols by comparing fissured-grain percentage before and after high-humidity fissure induction among 239 rice varieties. The percentage before fissure induction includes grains cracked pre-harvest (e.g. field fissuring) and post-harvest (e.g. during storage). Before fissure induction, none of the examined rice varieties had a fissured-grain percentage higher than 10% (A). Actually, 38.9% of the examined rice varieties had no fissured grains; another 52.72% had a fissured-grain percentage ranging from 0% to 5%; only 8.37% had a fissured-grain percentage higher than 5% lower than 10% (A and B). In Contrast, the fissured-grain percentage of every examined variety increased greatly after high-humidity induction and 97.91% showed a fissured-grain percentage higher than 10% (1A and B). These results demonstrate that when grains were prepared as we described, percentages of fissured grains before fissure induction are low enough and unlikely to interfere with the data after high-humidity induction, although not all of the rice varieties can avoid grain fissuring before the fissure-induction experiments. Thus, our data of fissured-grain percentages after induction were true reflections of grain fissure resistance.

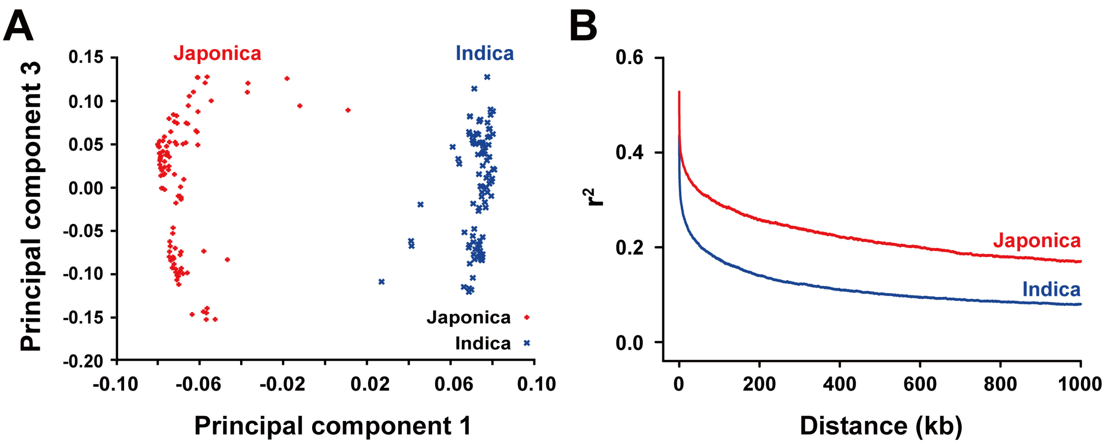

Fig. S2 The *Japonica* and *Indica* varieties used show rapid linkage disequilibrium decay

A. Principal component analysis of rice varieties.

B. Genome-wide averaged distance of linkage disequilibrium decay for *Japonica* and *Indica* varieties.

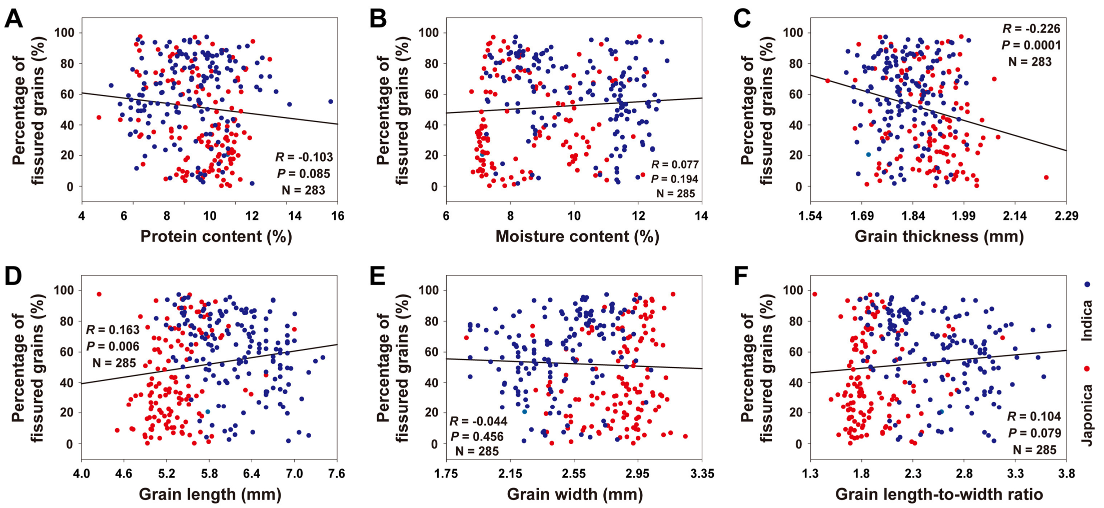

Fig. S3 No correlation between fissured-grain percentage and protein content, moisture content, or the thickness, length, width and length-to-width ratio of grains

A.Correlation analysis between fissured-grain percentage and protein content.

B. Correlation analysis between fissured-grain percentage and moisture content.

C. Correlation analysis between fissured-grain percentage and grain thickness.

D. Correlation analysis between fissured-grain percentage and grain length.

E. Correlation analysis between fissured-grain percentage and grain width.

F. Correlation analysis between fissured-grain percentage and grain length-to-width ratio.

R and *P* values were generated by Pearson correlation and N indicates the number of rice varieties used for analysis.

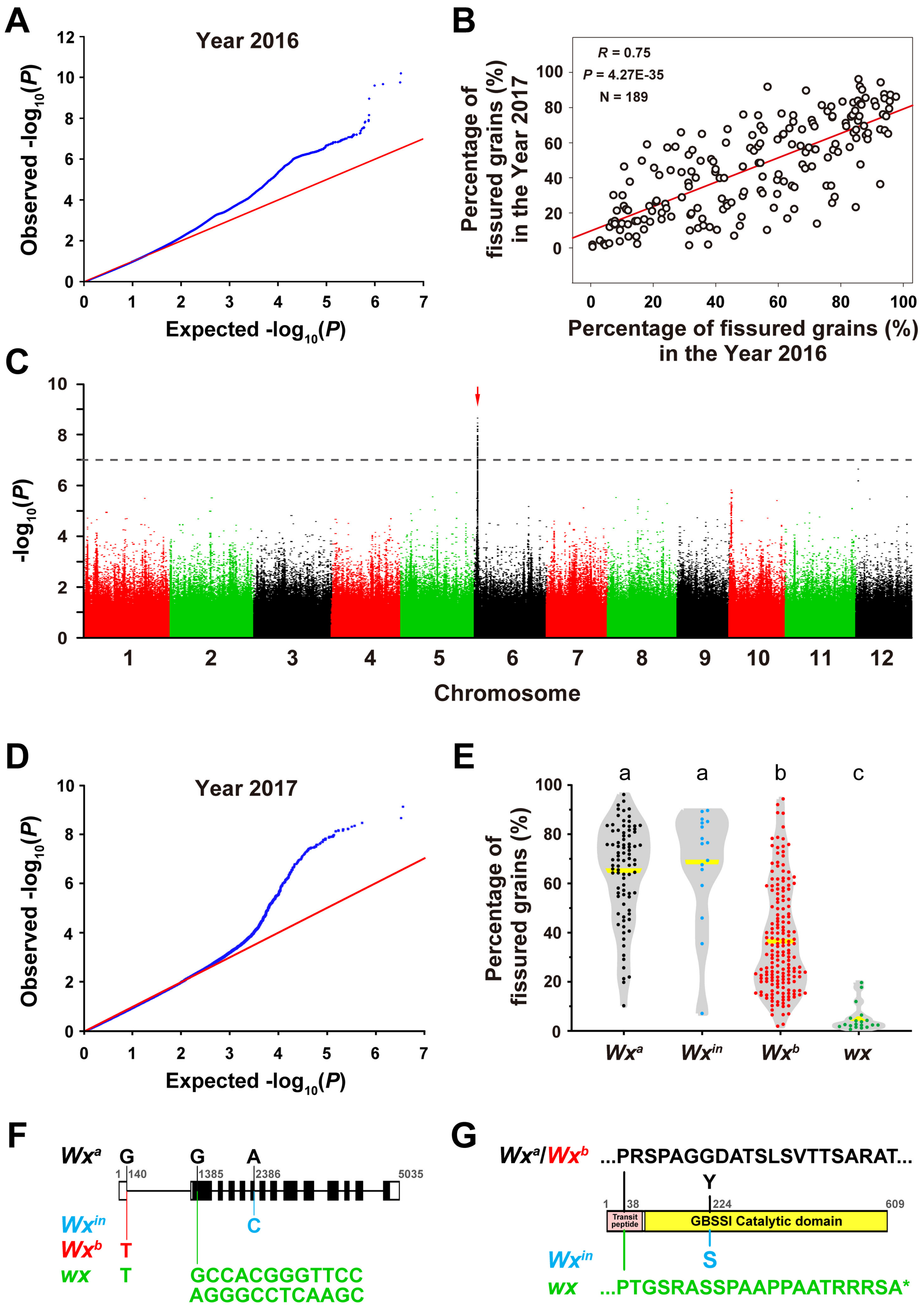

Fig. S4 Data for GWAS on grain fissure resistance

A. Quantile-quantile plot of GWAS on fissured-grain percentages evaluated in 2016.

B. Correlation between fissured-grain percentages evaluated in 2016 and 2017. Pearson correlation was used to generate R and *P* values and N indicates the number of rice varieties used in the correlation analysis.

C. Manhattan plot of GWAS for fissured-grain percentages assessed in 2017.

D. Quantile-quantile plot of GWAS on fissured-grain percentages assessed in 2017.

E. Distribution of fissured-grain percentages assessed in 2017 among rice varieties with different *Wx* alleles. Each dot on violin plots represents the mean value of two independent evaluations of one variety. Yellow lines on violin plots indicate the averages. Different letters indicate significant differences at $P<0.01$ (one-way ANOVA).

F. Genomic variations of Wx^{in}, Wx^{b} and *wx* compared with Wx^{a}. The numbers indicate positions on the genomic sequence of *Wx*. White boxes, black boxes and black lines represent the UTR regions, ORF and introns of *Wx*, respectively.

G. Variations in proteins encoded by Wx^{in} and *wx* compared with Wx^{a} and Wx^{b}. The numbers indicate positions on the amino acid sequence of GBSSI. Domains are shown in boxes with different colors.

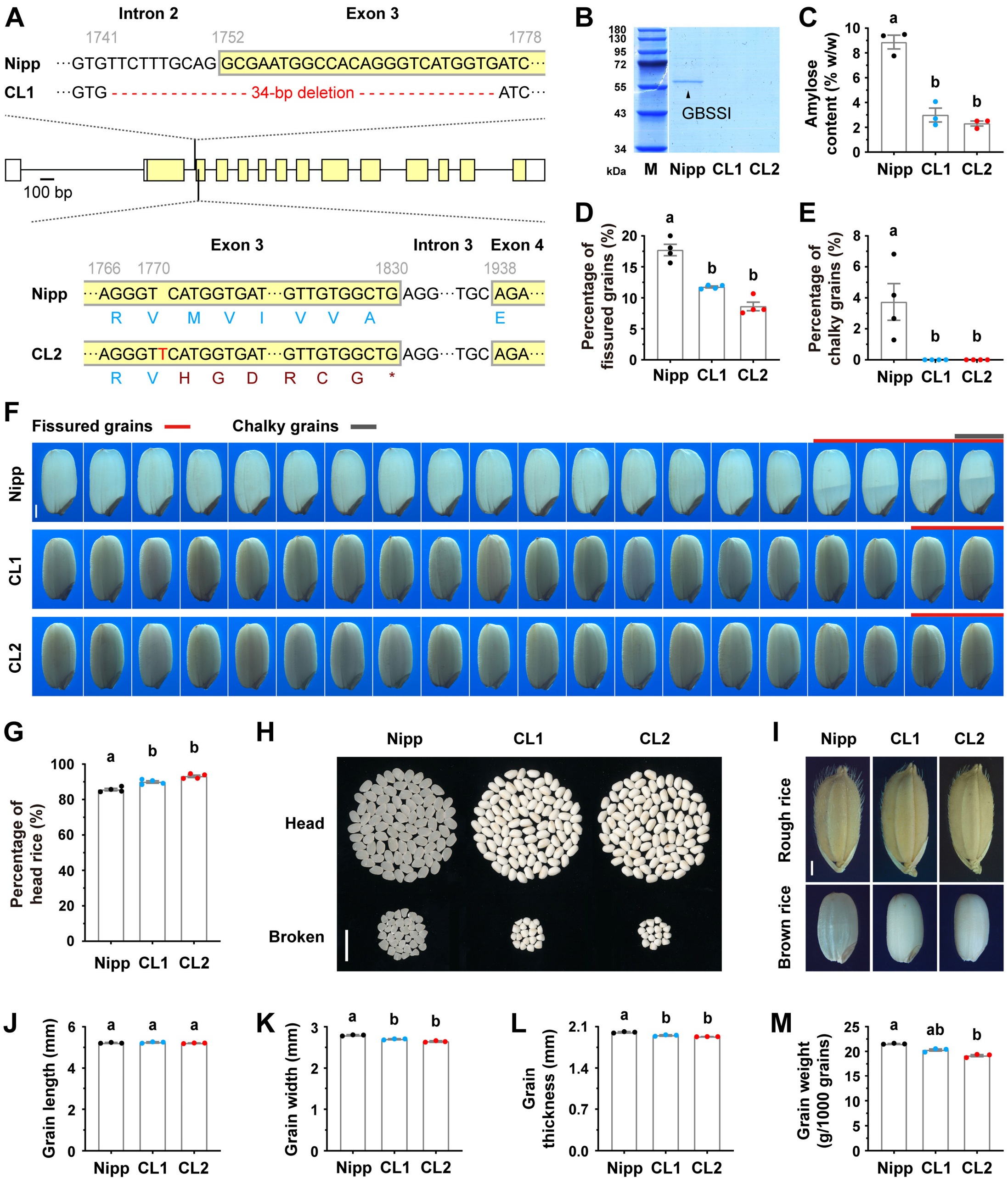

Fig. S5 CRISPR/Cas9-mediated knockout of Wx^b improves GFR and HRY

A. DNA mutations in CRISPR/Cas9-mediated Wx^b knockout lines (CL1 and CL2) under Nipponbare (Nipp) backgrounds. White boxes, yellow boxes and black lines indicate the 5′ and 3′ untranslated regions, open reading frames and introns, respectively. Nucleotides are numbered from the transcription start site (+1) and indicated by grey numbers. Red letters show the 34-bp deletion and the T insertion that lead to a splice junction mutation between the 2nd intron and the 3rd exon and a reading frameshift in the 3rd exon in CL1 and CL2, respectively. In CL2, blue and dark red letters indicate the normal and frameshift amino acids, respectively and the star indicates the premature translation termination.

B. Sodium dodecyl sulphate-polyacrylamide gel electrophoresis of the *Wx*-encoded GBSSI proteins, demonstrating *Wx* expression is absent in the two knockout lines.

C. The amylose content quantified by the lectin-binding method, showing the amylose content of starch in the two knockout lines were significantly lower than that in Nipponbare.

D. The fissured-grain percentages assessed after high-humidity treatment.

E. The percentages of chalky grains. Chalkiness in Nipponbare was very slight white-belly as shown in F.

F. Representative grain images showing differences in the percentages of fissured and chalky grains between Nipponbare and the two Wx^b knockout lines. Bar = 1 mm.

G. The head-rice percentages assessed after high-humidity treatment.

H. Separation of head and broken rice after 100 high-humidity-treated grains were dehulled and milled. Bar = 1 cm.

I. Mature grains. Bar = 1 mm.

J. Grain length.

K. Grain width.

L. Grain thickness.

M. Grain weight.

In C-E, G and J-M, Data are means ± SE (n = 3-4 tests) and different letters indicate significant differences at $P<0.01$ (one-way ANOVA).

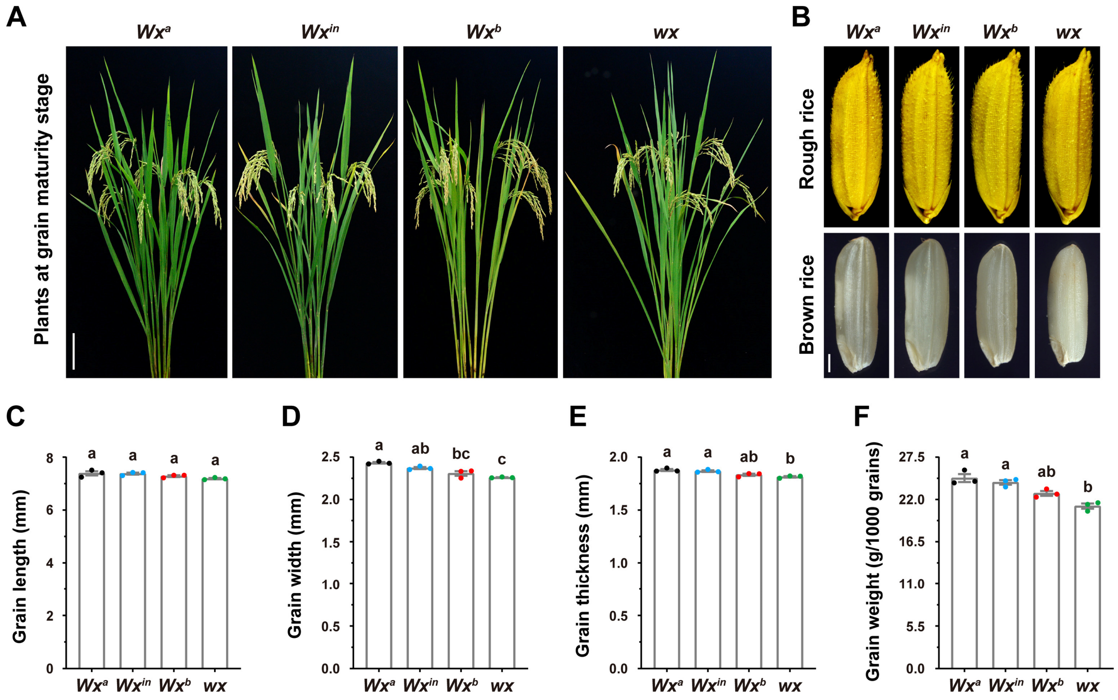

Fig. S6 Plant and grain phenotypes of *Wx* allelic variations

A. Plants at grain maturity stage. Bar = 10 cm.

B. Mature grains. Bar = 1 mm.

C. Grain length.

D. Grain width.

E. Grain thickness.

F. Grain weight.

In **C-G**, different letters indicate significant differences at $P<0.01$ (one-way ANOVA).

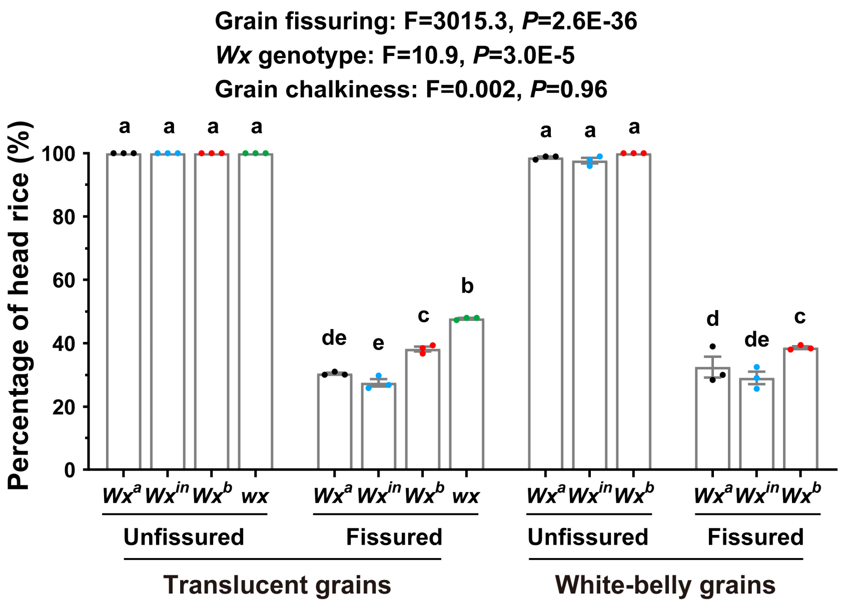

Fig. S7 Grain fissuring rather than chalkiness significantly reduces head rice yield

Head-rice percentages of translucent and white-belly grains with one fissure or without fissures (unfissured). NIL-wx was not included in the white-belly group due to its having almost no chalky grains. Data are means ± SE of triplicate assessments (at least 95 grains for each test). Different letters indicate significant differences at $P<0.01$ (one-way ANOVA). The F and P values were generated by multi-factor variance analyses.

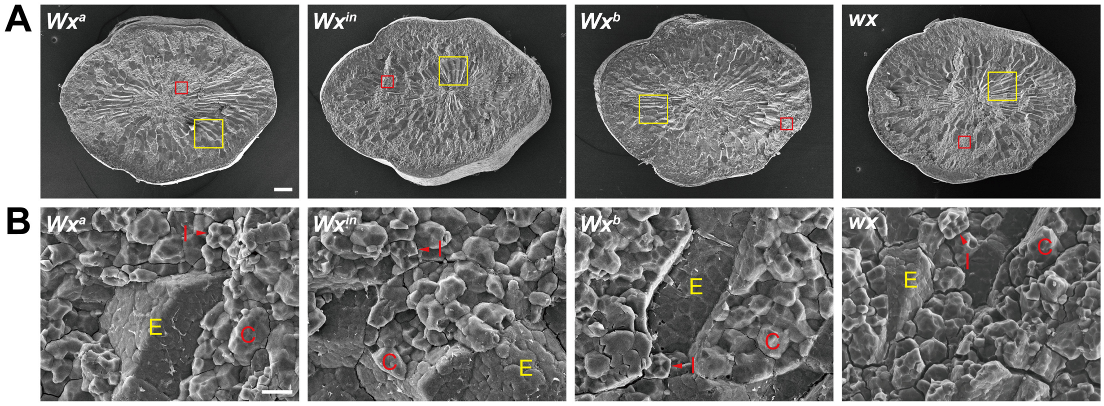

Fig. S8 Fissured grains of the four *Wx* NILs have similar morphology in fracture surfaces

A. Fracture surfaces of fissured grains observed by scanning electron microscopy. Smooth cell-shape surfaces and rough granular surfaces are exemplified in big yellow and small red boxes, respectively. Scale bar = 200 µm.

B. A magnified area of the grain fracture surfaces in panel A, showing smooth endosperm cell surfaces (yellow "E") and rough granular surfaces filled with exposed individual (red "I") or compound (red "C") starch granules. Scale bar = 10 µm. These endosperm cells are only residual cell corpses stuffed with storage products due to programmed cell death during grain filling.

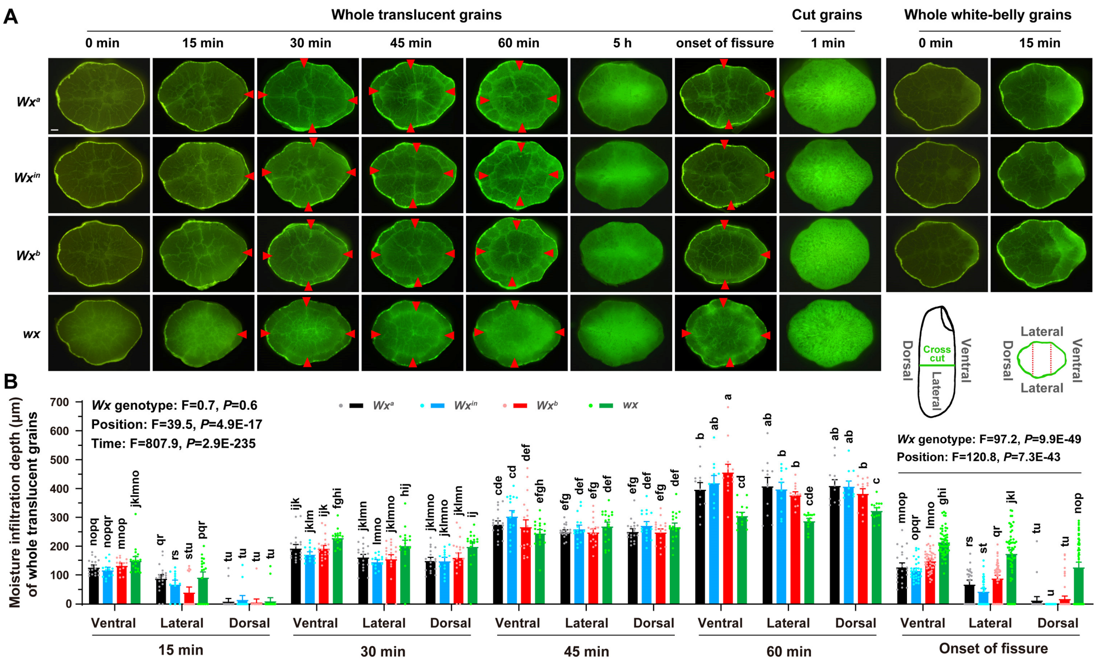

Fig. S9 Translucent grains of the four *Wx* NILs show similar moisture infiltration behaviors

A. Representative cross-sections of DTAF stained translucent and white-belly grains after water soaking for the indicated time. Red arrowheads mark the bright boundaries showing the depth of moisture infiltration. Scale bar = 200 μm. Dry and whole translucent grains could not be stained by DTAF and only showed background fluorescence (0 min), while crosscut translucent grains can be saturated with water immediately and dyed completely with DTAF (cut grains, 1 min), suggesting DTAF staining can be used as an imaging tool to examine moisture infiltration across grains.

B. Moisture infiltration depth of whole translucent grains in different grain parts at indicated soaking time. The diagrams in the upper right corner show the positions of different grain parts. Data are means ± SE (n = 12-22 grains). Different letters indicate significant differences at $P<0.01$ (one-way ANOVA). The F and P values were generated by multi-factor variance analyses.

This figure includes all data showing the complete process of moisture infiltration across grains at different time points, among which the data at important time points are shown in Figure 3A and B.

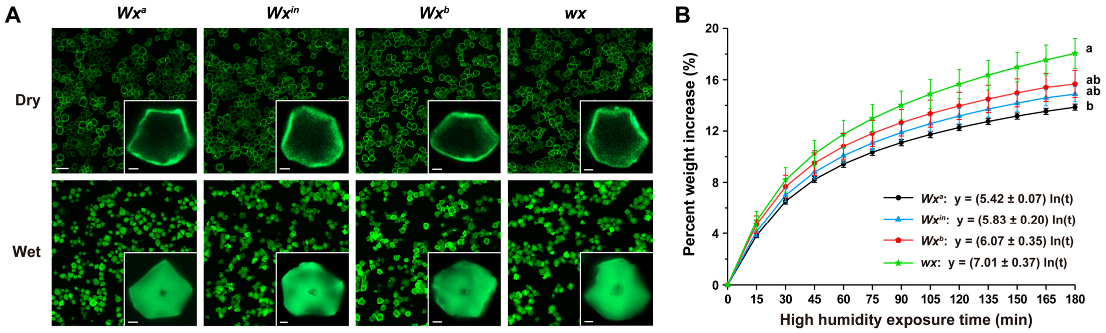

Fig. S10 Starch granules of the four *Wx* NILs show similar moisture absorption behaviors

A. DTAF staining of dry and wet starch granules. Scale bar = 10 μm. The insets show magnified single granules with 1-μm bars.

B. Percent weight increases of thin layers of starch granules at indicated moisture-treated time. Moisture absorption rates calculated from the logarithmic curves are shown in brackets after symbols. Data are means ± SE of triplicate analyses. Different letters indicate significant differences at $P<0.01$ (one-way ANOVA).

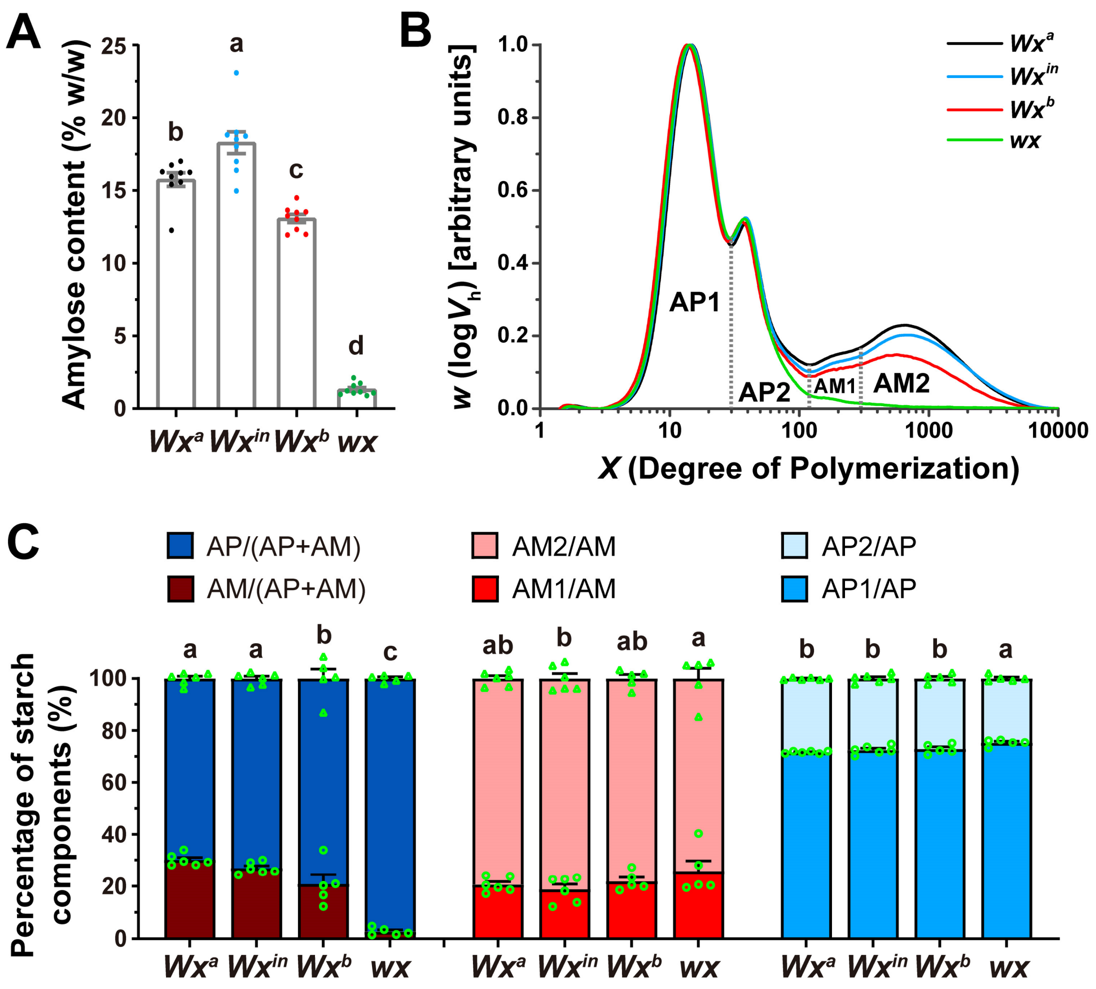

Fig. S11 The *Wx* NILs show variations in amylose content

A. The amylose content quantified by the lectin-binding method. Data are means ± SE (n = 9 tests).

B. Molecular weight distribution profiles of debranched starches revealed by SEC. The areas under the SEC curves are divided into four peaks, namely AP1 AP2, AM1 and AM2.

C. Percentages of starch components calculated from area proportions of different peaks under the SEC curves (n = 5-6 analyses). For example, AM/(AP+AM) indicates dividing the total area of all the four peaks by that of AM1 and AM2 peaks.

In **A** and **C**, significant differences are shown by different letters (P<0.01, one-way ANOVA).
