# Supplementary figures and images for: Waxy is an important factor for grain fissure resistance and head rice yield as revealed by a genome-wide association study
Source: J Exp Bot. 2022 Sep 2;73(19):6942–54. doi: 10.1093/jxb/erac330 (PMC9629786; doi:10.1093/jxb/erac330)

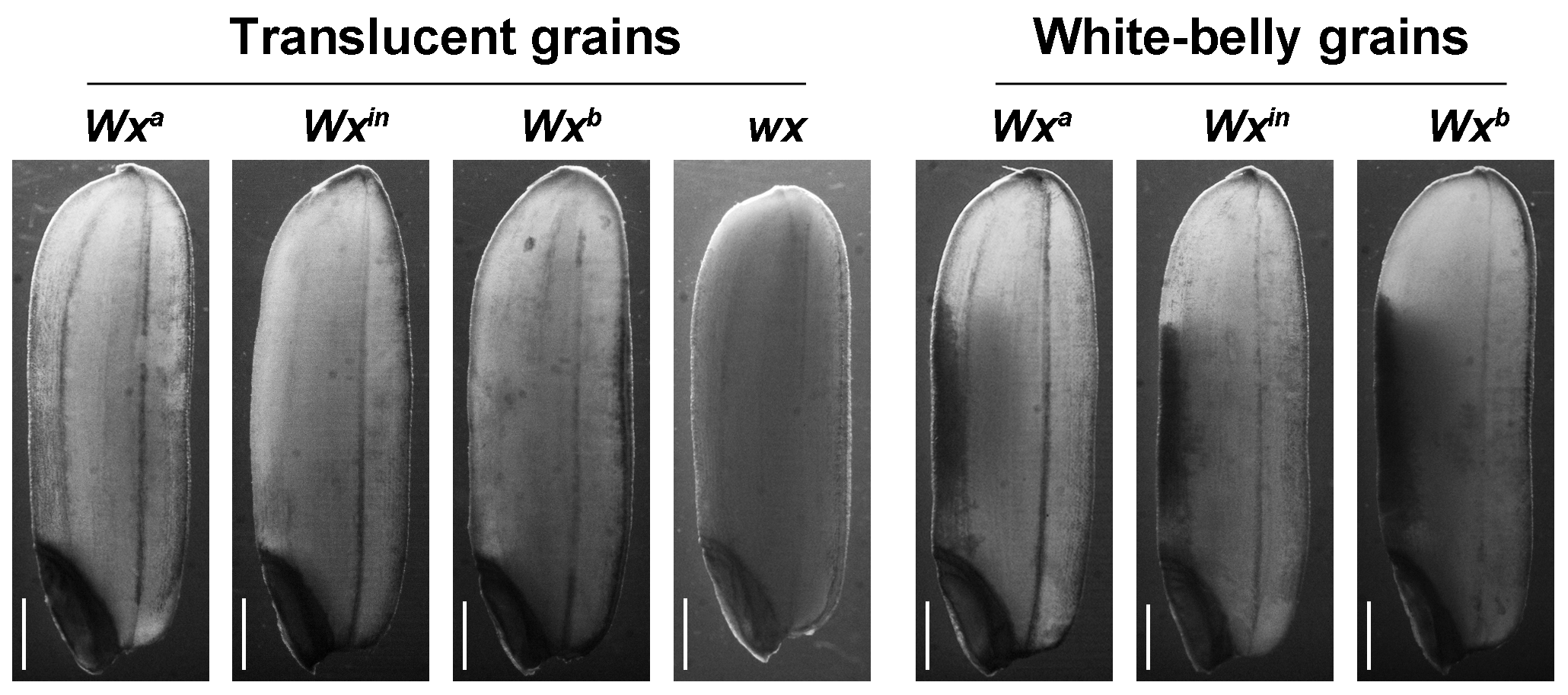

Supplement: erac330_suppl_Supplementary_Movie_S1 [file erac330_suppl_supplementary_movie_s1.gif]
